# Tennis Specialization and Consequence of Injury/Illness Following Retirement

**DOI:** 10.3390/sports11050106

**Published:** 2023-05-17

**Authors:** Ecaterina Vasenina, Jeffrey R. Stout, David H. Fukuda

**Affiliations:** Physiology of Work and Exercise Response (POWER) Laboratory, Institute of Exercise Physiology and Rehabilitation Science, University of Central Florida, Orlando, FL 32816, USA

**Keywords:** tennis, early specialization, OSTRC, HRQOL

## Abstract

(1) Background: There is a lack of literature that examines the impact of early vs. late sport specialization on quality of life after retirement from tennis. Thus, the purpose of this study was to examine the relationship between early specialization in the sport of tennis and health outcomes after retirement from collegiate/professional sport; (2) Methods: Participants were recruited through social media posts, newsletters, and contacts with tennis organizations. Basic demographic and injury information was collected from 157 former tennis players, along with the age of tennis specialization and two questionnaires: the Oslo Sports Trauma Research Center Questionnaire on Health Problems (OSTRC), and the CDC HRQOL-14 “Healthy Days Measure” Questionnaire (HRQOL); (3) Results: Significant differences (F_1,117_ = 5.160, *p* < 0.025) in the specialization age between the low (11.9 ± 4.5 y) and high (9.8 ± 4.1 y) OSTRC groups were found after covarying for the current age. No difference (F_1,72_ = 0.676, *p* < 0.414) was shown among the high (10.9 ± 4.4 y) and low (11.28 ± 4.6 y) HRQOL groups for the specialization age after covarying for the current age. A weak negative correlation was identified between the OSTRC score and specialization age (r = −0.233, *p* = 0.008), while no significant changes were shown between the specialization age and HRQOL score (r = −0.021, *p* = 0.857), or between the OSTRC and HRQOL scores (r = 0.146, *p* = 0.208); (4) Conclusions: Retired tennis players with low injury/illness severity scores specialized in tennis later than those with high injury/illness severity scores, while no differences in the specialization age were noted when the sample was separated into HRQOL groups.

## 1. Introduction

Tennis is a worldwide sport that is physically demanding when played at the professional level, with a high level of stress placed on muscles and joints through abrupt sprints and repetitive racquet motions [1]. While lifelong moderate exercise has been associated with numerous health benefits [2], participation at the elite level imposes high physical demands and increased levels of injuries [3]. It has been reported that professional athletes play through pain and injury instead of taking time to recover [4]. This practice may have negative health consequences later in life after their tennis careers are over. There is a lack of literature on the effects of early sport specialization in tennis; however, participation in any sport at the professional/collegiate level requires a high degree of time commitment and physical exertion. For example, Simon et al. [5] demonstrated that collegiate athletes experienced continued stress and are subjected to increased vulnerability to injury and overtraining, which, in turn, might negatively influence their quality of life later in life. Specifically, soccer players who experience knee injuries or dysfunction during their playing careers tend to experience a lower quality of life compared to non-athletes [6]. Additionally, former Division 1 athletes experience more limitations in their ability to maintain daily physical activity (i.e., getting to/from work) and/or exercise when compared to non-athletes [5].

Early sport specialization typically refers to individuals who specialized in a single sport “before the age of 12” [7], and it has been assumed to be associated with an increased likelihood of competing at a higher level. This assumption is largely based on the athletes from Eastern European countries who were praised by the media for their success in sports at a very young age [8]. Interestingly, the effectiveness of early sport specialization and its effect on success in professional sport has recently been questioned. In their 2016 consensus statement, the American Orthopedic Society for Sports Medicine concluded that young children who specialize early in their sport are more likely to experience overuse injuries and burnout than children participating in multiple sports [9]. Furthermore, in their 2014 study of professional baseball players, Ginsburg et al. [10] surveyed 708 minor league professional baseball players. The authors concluded that athletes who specialized in baseball after the age of 12 were more likely to receive collegiate scholarships than athletes who specialized in a single sport at an earlier age. Furthermore, a study on junior tennis players showed that the players who specialized only in the sport of tennis were more likely to report injuries compared to athletes who specialized in multiple sports [11]. While early specialization in a single sport is commonly viewed as a necessary aspect of an athlete’s success, it might not be required to achieve high competitive levels in a professional sport and could lead to negative health and injury consequences [12].

These negative consequences of early specialization might be linked to the high demands that a sport places on an individual. When a junior player is not fully physically developed, the combination of high-intensity training and non-sufficient recovery between training sessions might lead to a higher occurrence of injuries [13]. Additionally, a higher number of injuries could result in a lower quality of life after retiring from the sport. For example, Moreira et al. [14] reported an association between the high number of sports injuries in basketball master athletes and the reduced dimensions related to health assessed through the physical, mental, and social symptoms reported by the athletes. Furthermore, McDonald et al. [15] reported that elite wrestlers who specialized prior to age 12 sustained a greater number of serious injuries compared to those who specialized after the age of 12.

Jayanthi et al. [11] collected surveys from 540 junior tennis players and monitored their progress through a 4-week period during the summer tournament season in the year of 2008. They found that players who specialized early in tennis were more likely to have reported tennis-related injuries in the previous year compared to players who specialized later. Furthermore, Sinkovic et al. [16] examined the effect of biological age on speed-explosive properties in young tennis players and found that tennis players of older biological age achieve better results in the variables of speed, agility, and explosive power when compared to players of younger biological age. Interestingly, there is a lack of literature that examines the impact of early vs. late sport specialization on quality of life after retirement from tennis. There is currently no research looking at the effects of early specialization, specifically in the sport of tennis, and its effect on health outcomes after retirement from a collegiate/professional sport. Thus, this study will examine the relationships between early specialization in the sport of tennis and health outcomes following retirement in collegiate/professional athletes. We hypothesize that former professional and collegiate tennis athletes who experience poorer health outcomes following retirement specialized earlier in the sport compared to the ones who experience better health outcomes.

## 2. Materials and Methods

### 2.1. Procedure

This study utilized a cross-sectional retrospective analysis. A combination of convenience and snowball sampling was used to recruit participants through social media posts, newsletters, and contacts with tennis organizations, such as the International Tennis Performance Association and Intercollegiate Tennis Association. The tennis organizations distributed the link to the survey to potential participants through newsletters and social media posts. After choosing to click on the link to participate in the study, participants were directed to the page that included the consent form. After reading and signing the consent form, participants were directed to the survey. All data collection was computer-based. For the purpose of this study, participants were excluded from analysis if they were younger than 18 years old, did not play collegiate (Division 1) or professional tennis, and/or if they were retired from collegiate/professional tennis for less than 1 year. There were no other inclusion or exclusion criteria that would prevent participants from completing the survey.

The study was approved with an exempt determination by the university’s Institutional Review Board (STUDY00003714) and took place January through August of 2022. The anonymous survey was conducted using a web-based survey system (Qualtrics, Provo, Utah) and consisted of four questionnaires: basic demographic and injury questionnaires, the Oslo Sports Trauma Research Center Questionnaire on Health Problems (OSTRC) [17], and the CDC HRQOL-14 “Healthy Days Measure” Questionnaire (HRQOL) [18].

### 2.2. Participants

A total number of 224 responses was received from former Division 1 collegiate tennis players or professional tennis players who were retired from the sport (did not play any tournaments) for at least one year. Sixty-seven responses were removed from all analyses due to not meeting the inclusion criteria or for providing incomplete responses to either the HRQOL or OSTRC portions of the survey. Thus, data from 157 retired athletes were considered. Only 75 out of 157 participants fully completed the HRQOL portion of the questionnaire and listed their age and years since retirement from the sport (Table 1), while only 120 out of 157 participants fully completed the OSTRC portion of the questionnaire and listed their age and years since retirement from the sport (Table 2).

### 2.3. Demographic Information, Injuries, and Early Specialization

Basic demographic and injury information was self-reported and collected along with the age at tennis specialization. Demographic information included questions about biological sex, country of origin, years played on the professional tour (if they did play on the tour), years since retirement from professional/collegiate tennis, and highest achieved tennis ranking (both singles and doubles) in collegiate/professional tennis and/or both. The injury questionnaire was adapted from the survey by Rugg et al. [19] and includes 6 questions, with 2 questions appearing only if a certain answer is selected on the previous question. Specifically, if the former athlete indicates that they experienced severe injury that required them to miss competition for more than 30 days/ended their season, then information about the location of that injury will be obtained. Similarly, if the former athlete indicates experiencing a sport-related injury that required surgery, then information about the location of that surgery will be obtained. Finally, the early-specialization questions were adapted from an early-specialization survey conducted by Rugg et al. [19] and focused on whether former athletes participated in more than one organized sport between the ages of 5 and 18, whether they played more than one sport until the end of high school, and at what age they began to participate in a single sport.

### 2.4. Quality of Life and Injury/Illness Severity Scores

The previously validated OSTRC [17] recorded the magnitude, symptoms, and consequences of overuse injuries and illnesses that participants experienced in the last 7 days resulting in a current injury/illness severity score. Based on their responses, participants were divided into low (scores of 0–49) and high (scores of 50–100) OSTRC groups, with higher scores representing greater injury/illness severity scores. The HRQOL [18] measured physical and mental health preconceptions (e.g., energy level, social support, and socioeconomic status), specifically focusing on the sum of physically and mentally unhealthy days experienced during the past 30 days. Based on their responses, participants were divided into high (scores of 0–15) and low (scores of 16–30) HRQOL groups. High scores represented more unhealthy days and, thus, lower quality of life. The maximum achievable scores were 60 for the HRQOL and 100 for the OSTRC, with zero being the minimum score for both measures.

### 2.5. Data Analysis

Descriptive statistics, including minimum and maximum values and 95% confidence intervals, were generated. Two one-way ANCOVAs were performed to compare the differences in the specialization age between the high and low HRQOL and OSTRC groups while controlling for age. Levene’s test indicated that all dependent variables met the assumption of equal variances (*p* = 0.26). Normality of the residuals was evaluated through visual examination of Q–Q plots. Partial n^2^ was used to determine effect sizes, and the values were interpreted as small effect (0.01), moderate effect (0.06), and large effect (0.14) [20]. Pearson’s r correlations were used to examine relationships between the HRQOL score, OSTRC score, and single-sport specialization age. Correlation coefficients (r^2^) were interpreted as weak (0.01–0.39), moderate (0.40–0.69), and strong (0.70–1.00) [21]. JASP (version 17.1, Amsterdam, The Netherlands) [22] was used for statistical analysis, with the significance level set at <0.05. Values are reported as means and standard deviations, unless otherwise noted.

## 3. Results

The age of all the participants included in the analysis (*n* = 157) varied between 20 and 82 years old, with a mean age of 40.7. Only 151 participants indicated years since their retirement from playing collegiate tennis. This number ranged from 61 years to 1 year, with a mean of 18.5 years. All 157 participants indicated their specialization ages, which ranged from 5 to 18 years, with a mean of 11 years old. Finally, there was a variety of rankings among the participants. The highest professional singles ranking was #4 in the world, with a low ranking of 1753 and mean ranking of 696, with 51 participants listing their rankings. The highest professional doubles ranking was #1 in the world, with a low ranking of 1753 and mean ranking of 613, with 44 participants listing their rankings. Further, the highest collegiate singles ranking was #1, with a mean ranking of 55.4, and with 53 participants indicating their rankings. The highest collegiate doubles ranking was #1, with a mean ranking of 41.7, and with 53 participants indicating their rankings. Finally, the highest collegiate team ranking was #1, with a mean ranking of 24.9, and with 116 participants indicating their rankings.

Significant differences (F_1,117_ = 5.160, *p* = 0.025, partial n^2^ = 0.042) in the specialization age between the low (11.9 ± 4.5 y) and high (9.8 ± 4.1 y) OSTRC groups were found after covarying for the current age. Levene’s test indicated equal variances (F = 0.033, *p* = 0.855). No difference (F_1,72_ = 0.676, *p* < 0.414, partial n^2^ = 0.009) was shown among the high (10.9 ± 4.4 y) and low (11.28 ± 4.6 y) HRQOL groups for the specialization age after covarying for the current age. Levene’s test indicated equal variances (F = 0.432, *p* = 0.513). A weak negative correlation was identified between the OSTRC score and specialization age (r = −0.233, *p* = 0.008), while no significant correlation was shown between the specialization age and HRQOL score (r = −0.021, *p* = 0.857), or between the OSTRC and HRQOL scores (r = 0.146, *p* = 0.208).

## 4. Discussion

The present manuscript examined the relationship between early specialization in the sport of tennis and health outcomes after retirement from a collegiate/professional sport. Our primary findings were that tennis players with low injury/illness severity scores specialized later than those with high injury/illness severity scores. When the sample was separated into HRQOL groups, there were no differences in age by specialization. In addition, a weak negative correlation between the OSTRC scores and age of specialization was observed. These findings suggest that injury/illness severity scores are associated with the age of sport specialization, whereas quality-of-life scores may not be associated with the age at which an athlete specialized in tennis. Our initial hypothesis stated that former professional and collegiate athletes who specialized early in the sport of tennis will experience lower health outcomes compared to the ones that specialized in tennis later on. Based on the results of the study, our hypothesis was partially accepted. Tennis athletes who reported higher injuries/illness scores after retirement specialized earlier in the sport of tennis than those who reported lower injury/illness scores, while there were no differences in the specialization age when examining the quality-of-life scores.

Our results on the effect of sport specialization on health outcomes after retirement are in agreement with previous research on sport specialization in athletes. Croci et al. [23] found that baseball players who specialized early in their sport were more likely to have lower throwing-arm function and a higher likelihood of injuries, while Jayanthi et al. [11] reported a higher number of wrist injuries in elite junior tennis players who specialized only in tennis. Tennis is an asymmetrical sport that requires many one-sided movements, such as serving and hitting forehands and backhands. This results in a heavy load placed on the dominant side of the body [24] and early bilateral differences, such as greater dominant wrist flexion and extension strength, which have been observed in young elite-level female tennis players [25]. In addition to asymmetry, other physiological consequences of early specialization were reported that include issues with developing knees and increased inflexibility around the knee joint [26]. Thus, when junior athletes are not fully developed, the lack of sufficient recovery between sessions, in addition to the high intensity during training sessions, might lead to a higher number of injuries. In addition, the amount of time athletes spend daily on the tennis court could result in the development of asymmetry, which, in turn, could increase the mechanical loading on the dominant side of the body and result in overuse injuries.

This level of asymmetry is attributed to the high level of mechanical loading placed on the dominant extremities while performing tennis strokes [27]. For example, a study by Rynkiewicz et al. [28] examined the degree of muscle mass asymmetry and its association with the dominant upper limbs in 16 active tennis players (15 right-handed and 1 left-handed) and compared the results to a control group consisting of 14 right-handed and 2 left-handed participants. The authors assessed body composition via multi-frequency bioelectrical impedance measurements and discovered that there were significant muscle mass differences in the dominant limb compared to the non-dominant one. Interestingly, the control group was characterized as being lower than the tennis player group in terms of muscle mass distribution in the upper limbs [28]. Being a consequence of tennis, asymmetry could result in improper body stature and potentially lead to imbalances in the skeletal structure [26]. These consequences could result in players eventually experiencing joint overloading and injuries.

High levels of mechanical loading could lead to overuse injuries. For example, it has been observed that repetitive loading of the wrist and forearm might result in the development of humeral epicondylitis [25]. Additionally, tennis involves a number of lateral movements as well as quick changes in direction, which could put significant stress on the hips, knees, and ankles [1]. Thus, the potential overuse injuries and asymmetry that tennis players experience throughout their tennis careers could be related to early specialization in tennis and are in alignment with our results that showed an association between injury/illness severity scores and the age of sport specialization.

The OSTRC Questionnaire used in our survey recorded the magnitude, symptoms, and consequences of overuse injuries and illnesses that participants experienced in the last 7 days resulting in a current injury/illness severity score. Although the OSTRC Questionnaire was originally used as a monitoring tool to evaluate athletes’ health trends [17], it has also been utilized to determine the retrospective severity of illnesses and injuries [29,30]. The significant difference (*p* < 0.025) in the specialization age between the low and high OSTRC groups found in this study could be attributed to early specialization and the potential for its association with intense training. As reported by Jayanthi et al. [31], there is a risk that comes with early specialization and intense single-sport training that includes higher levels of injuries, psychological stress, and burnout. Furthermore, it has been reported that athletes at higher competitive levels experience higher rates of injuries [32], and there is an elevated risk of fractures and injuries during the peripubertal stage [33].

Although both the OSTRC and HRQOL questionnaires measured the physical health of individuals that included illness and injury, the HRQOL questions also focused on mental health preconceptions (e.g., stress, depression, and problems with emotions) while specifically focusing on the sum of physically and mentally unhealthy days experienced over the past 30 days. Interestingly, sport participation has been associated with improved mental health, reduced stress, and enhanced positivity [34,35]. Athletes might struggle psychologically when transitioning out of professional/collegiate sport, but, overall, former athletes tend to show better vitality, emotional functioning, social role functioning, and mental health on the HRQOL Questionnaire [36]. Because the HRQOL Questionnaire is a sum of both physical and mental health problems, it is plausible to suggest that even if athletes experienced physically unhealthy days, their mental health scores remained elevated, resulting in an overall positive outcome related to quality of life.

### Limitations

All data received from the questionnaires were self-reported. While there is evidence to suggest that individuals are capable of reporting reliable information [37], it would have been ideal to follow up with the participants and double-check their reported information, in addition to asking them whether they obtained physicians’ diagnoses. In addition, some of these participants completed the survey years after participating in collegiate/professional tennis. There might have been recall bias and other confounding factors, including aging, employment status, and current socioeconomic status, that influenced the responses to questions related to specialization age, ranking, numbers of injuries, etc. [5].

Only 75 of 157 participants completed the HRQOL portion of the questionnaire, while 120 participants completed the OSTRC portion. The HRQOL portion of the questionnaire asked participants to enter numerical values, while the OSTRC portion provided four multiple-choice answers per question. It is possible that the participants had an easier time selecting a multiple-choice answer than typing a numerical value, which resulted in them skipping the HRQOL portion of the questionnaire.

Finally, some caution may be warranted with regard to the use of the OSTRC Questionnaire to determine injuries and illnesses at a single point in time due to it having been developed primarily as a monitoring tool via weekly sampling.

## 5. Conclusions

Sport participation can play an important role in youth development, but long-term consequences are rarely considered. On the one hand, based on our findings, it is plausible to suggest that early specialization in the sport of tennis might come with adverse consequences when it comes to the OSTRC scores, but it has limited impact on the HRQOL scores. On the other hand, later specialization in tennis might result in better OSTRC scores. Overall, it seems that participation in multiple sports early in life and the delay of early specialization in a single sport might support motor-skill-deficit reduction and help reduce the likelihood of sport-related injuries [38].

No differences in the specialization age were noted when the sample was separated into HRQOL groups. There was no correlation between the HRQOL and OSTRC scores. While both the OSTRC and HRQOL questionnaires measured the physical health of participants, only the HRQOL Questionnaire also measured their mental health. Based on previous research [36,37], participation in sports has been associated with improved mental health and increased positivity. Higher scores on the mental health component of the HRQOL Questionnaire could compensate for lower scores on the physical health component.

The findings of the current study could be applied by coaches and relevant stakeholders from a long-term athlete development perspective. However, prospective analyses as well as further work related to specific mechanisms leading to post-career injury/illness severity (i.e., sport-specific asymmetries, etc.) and the differentiation between collegiate and professional experience are still needed.

## Figures and Tables

**Table 1:** Survey response data for the overall, low, and high CDC HRQOL-14 “Healthy Days Measure” Questionnaire (HRQOL) groups.

	Overall (*n* = 75)					Low HRQOL (*n* = 61)					High HRQOL (*n* = 14)				
	Mean	±	SD	Lower 95% CL	Upper 95% CL	Mean	±	SD	Lower 95% CL	Upper 95% CL	Mean	±	SD	Lower 95% CL	Upper 95% CL
HRQOL Score	14.2 ± 13			11.2	17.1	9.2 ± 7.1			7.4	10.9	35.8 ± 4.4			30.6	41
Specialization Age (y)	11.0 ± 4.4			10.0	12.1	10.9 ± 4.4			9.8	12.08	11.3 ± 4.6			8.8	13.6
Current Age (y)	39.5 ± 15.0			36.3	42.8	40.7 ± 14.7			37.0	44.4	34.5 ± 13.0			27.8	41.2
Years Since Retirement	16.7 ± 14.0			13.6	19.8	17.5 ± 14.0			14.0	21.0	13.0 ± 12.0			12.4	6.5

SD: standard deviation, CL: confidence limit.

**Table 2:** Survey response data for the overall, low, and high Oslo Sports Trauma Research Center Questionnaire on Health Problems (OSTRC) groups.

	Overall (*n* = 120)					Low OSTRC (*n* = 89)					High OSTRC (*n* = 31)				
	Mean	±	SD	Lower 95% CL	Upper 95% CL	Mean	±	SD	Lower 95% CL	Upper 95% CL	Mean	±	SD	Lower 95% CL	Upper 95% CL
OSTRC Score	28.9 ± 34.6			22.7	35.1	10.9 ± 15.9			7.5	14.2	80.6 ± 16.3			74.9	86.3
Specialization Age (years)	11.4 ± 4.5			10.6	12.2	11.9 ± 4.5			11	12.9	9.8 ± 4.1			8.3	11.2
Current Age (years)	41.5 ± 14.5			38.9	44.1	42.1 ± 15.2			38.9	45.2	39.9 ± 12.6			35.5	44.4
Years Since Retirement	18.4 ± 13.9			15.8	20.8	18.7 ± 14.6			15.6	21.7	17.5 ± 12.2			13.2	21.8

SD: standard deviation, CL: confidence limit.

## Data Availability

Data are available upon request.
